# Development of the CHARIOT Research Register for the Prevention of Alzheimer’s Dementia and Other Late Onset Neurodegenerative Diseases

**DOI:** 10.1371/journal.pone.0141806

**Published:** 2015-11-23

**Authors:** Mark E. Larsen, Lisa Curry, Nikolaos Mastellos, Catherine Robb, Josip Car, Lefkos T. Middleton

**Affiliations:** 1 Global eHealth Unit, School of Public Health, Imperial College London, London, United Kingdom; 2 Neuroepidemiology and Ageing Research Unit, School of Public Health, Imperial College London, London, United Kingdom; 3 Lee Kong Chian School of Medicine, Nanyang Technological University, Singapore; University Of São Paulo, BRAZIL

## Abstract

**Background:**

Identifying cognitively healthy people at high risk of developing dementia is an ever-increasing focus. These individuals are essential for inclusion in observational studies into the natural history of the prodromal and early disease stages and for interventional studies aimed at prevention or disease modification. The success of this research is dependent on having access to a well characterised, representative and sufficiently large population of individuals. Access to such a population remains challenging as clinical research has, historically, focussed on patients with dementia referred to secondary and tertiary services. The primary care system in the United Kingdom allows access to a true prodromal population prior to symptoms emerging and specialist referral. We report the development and recruitment rates of the CHARIOT register, a primary care-based recruitment register for research into the prevention of dementia. The CHARIOT register was designed specifically to support recruitment into observational natural history studies of pre-symptomatic or prodromal dementia stages, and primary or secondary prevention pharmaceutical trials or other prevention strategies for dementia and other cognitive problems associated with ageing.

**Methods:**

Participants were recruited through searches of general practice lists across the west and central London regions. Invitations were posted to individuals aged between 60 and 85 years, without a diagnosis of dementia. Upon consent, a minimum data set of demographic and contact details was extracted from the patient’s electronic health record.

**Results:**

To date, 123 surgeries participated in the register, recruiting a total of 24,509 participants–a response rate of 22.3%. The age, gender and ethnicity profiles of participants closely match that of the overall eligible population. Higher response rates tended to be associated with larger practices (r = 0.34), practices with a larger older population (r = 0.27), less socioeconomically disadvantaged practices (r = 0.68), and practices with a higher proportion of White patients (r = 0.82).

**Discussion:**

Response rates are comparable to other registers reported in the literature, and indicate good interest and support for a research register and for participation in research for the prevention of age-related neurodegenerative diseases and dementia. We consider that the simplicity of the approach means that this system is easily scalable and replicable across the UK and internationally.

## Introduction

There are currently more than 35 million people worldwide living with dementia and, due to the ageing world population, this number is expected to double by 2030 and more than triple by 2050 to exceed 115 million [1]. The total cost of the management of dementia was estimated to be US$604 billion in 2010 [2] and in addition to the increasing morbidity, a recent study has shown that mortality associated specifically with Alzheimer’s dementia (AD) may be higher than previously thought [3].

In order to effectively address this major health and socioeconomic challenge, public funders and charities worldwide have identified research into causative pathways and risk factors for dementia, as well as drug discovery and development of new and effective disease-modifying therapies, as a high research priority [4]. The licensing of cholinesterase inhibitors in the late 1990’s for the symptomatic treatment of Alzheimer’s dementia, together with initial publications unravelling the role of the amyloid cascade and tau, were the principal catalysts in an enthusiastic wave of drug discovery and development towards targeted disease-modifying therapies for this devastating condition. More than a decade later, these attempts have proven disappointing as new compounds, typically tested in patients at an advanced stage of Alzheimer’s dementia, have failed at various stages of development–with a failure rate of 99.6% of potential agents [5]. Coupled with these disappointing results, there is now recognition that the AD-type pathological changes start accumulating years, if not decades, prior to the onset of dementia. In predominantly inherited familial AD, it was estimated that Alzheimer’s dementia pathology, such as amyloid deposition, initiates decades prior to the projected date of disease onset and that some evidence of cognitive changes is detected years, if not a decade, prior to a dementia diagnosis [6].

It has been estimated that delaying the onset of Alzheimer’s dementia by one or two years could decrease global disease burden in 2050 by 11.8 million or 22.8 million cases, respectively [7]. The emphasis of clinical research has, therefore, begun to shift towards the early clinical phase of “mild cognitive impairment” (MCI) and even earlier, involving *at-risk* individuals in the pre-clinical, thus “prodromal Alzheimer’s dementia”, phases. This allows for primary or secondary prevention, as well as for disease modification [8] as later stages of established disease brain pathology may be too advanced to allow any disease modification or effective reversal of symptoms. In recent years, there have been several attempts to define this pre-dementia “prodromal AD” stage, both in its pre-clinical [9, 10] and early symptomatic (or “MCI due to AD”: MCI-AD) [9, 11] stages, mostly relying on imaging and biomarker data derived from the analyses of cerebrospinal fluid studies. These criteria, which still require *real-life* validation, are mainly intended for clinical research purposes and are not currently applicable in the general population as they are based on technologies and methods that are not easily accessible.

This prodromal population will largely be unknown to specialist services and therefore would be suitable for identification from primary care or in the general population. However, it is widely acknowledged that recruiting individuals into clinical trials is a particularly challenging endeavour [12–15], with 45% of studies recruiting less than their target and 54% of trials requiring an extension to fulfil recruitment obligations [16]. Recruitment in primary and secondary care depends on the active engagement of clinicians to recruit eligible individuals and often fails to meet the expected targets due to time-pressure in a busy consultation schedule. Primary care clinicians may also feel that they lack the skills or confidence to give an effective explanation of a clinical research study. Furthermore, there is recognition that particular groups of individuals are more challenging to recruit into studies, including young men, the elderly, those with mental health problems or patients who are socially deprived [17–19]. Prevention studies recruiting healthy volunteers require much larger sample sizes than treatment trials [20, 21] in order to demonstrate drug efficacy and require different recruitment strategies, going beyond the traditional “specialised clinic” model. In the USA, Medicare lists or voter registration rolls have been used to aid recruitment [22, 23], however, such recruitment strategies may not always be suitable due to ethical and practical considerations. Recruitment into prevention studies may be improved if registers of individuals who are specifically interested in such studies are available.

Research registers connect potential research participants with research studies, thereby enabling researchers to successfully recruit large numbers of suitable, motivated participants and overcome some of the barriers encountered when using traditional methods of recruitment into clinical trials. Such registers have successfully been used to recruit into treatment trials for dementia [24] and the Banner Alzheimer’s Institute has initiated a USA-based register of individuals interested in learning about prevention studies [25]. We sought to leverage the prominent and well-structured role of primary care within the National Health Service (NHS) in the United Kingdom to develop a primary care based register to address the growing need for recruitment for clinical studies (including trials) of late-onset dementias. With this in mind, we established the CHARIOT register (an acronym for Cognitive Health in Ageing Register: Investigational, Observational and Trial studies in dementia research) as a collaboration between general practices and researchers within the School of Public Health at Imperial College London. The CHARIOT register was designed specifically to support recruitment into observational or interventional studies for primary prevention trials or secondary prevention strategies for dementia and other cognitive problems associated with ageing.

## Methods

This project was approved by the National Research Ethics Service (NRES) East Midlands–Derby 1 committee. To join the CHARIOT register, the invitee signed both copies of an informed consent form and returned these to the register team in a pre-paid reply envelope. The register has developed in three distinct phases: the initial pilot phase, to establish the feasibility and levels of interest in the register; the optimisation phase, during which the operation of the register was iteratively adapted to maximise response; and the main recruitment phase.

### Recruitment

General practices across the London region were invited to participate in the CHARIOT register, and were offered a reimbursement for their involvement. Eligible participants were identified through searches of general practice records, identifying individuals aged between 60 and 85 and excluding those with a diagnosis of dementia (see Table 1 for an overview of inclusion/exclusion criteria, and S1 File for the complete list) or those whom the GP or practice staff considered would be inappropriate to contact. These exclusion criteria follow national guidelines for identifying patients with a diagnosis of dementia [26]. Patients with early signs of dementia were considered eligible for joining the register, and were not specifically excluded. Patients with other chronic health conditions were also considered eligible.

**Table 1:** List of inclusion/exclusion criteria.

Inclusion Criterion	Exclusion Criteria
Individual is aged 60–85 (inclusive) on the date of search	Individual has a recorded diagnosis of dementia:
	• Senile and pre-senile organic psychotic conditions
	• Other alcoholic dementia
	• Drug-induced dementia
	• Dementia in conditions classified elsewhere
	• Dementia in Alzheimer's disease
	• Vascular dementia
	• Dementia in other diseases classified elsewhere
	• Delirium superimposed on dementia
	• Alzheimer's disease
	• Pick's disease
	• Senile degeneration of brain
	• Dementia with Lewy bodies (DLB)

Invitation packs containing an invitation letter, a participant information sheet and two copies of an informed consent form were printed on GP letter-headed paper and posted to each invitee at their home address. To join the CHARIOT register, the invitee signed both copies of the informed consent form and returned these to the register team in a pre-paid reply envelope. Both consent forms were countersigned by a register investigator, with one copy being returned to the participant by post, one copy being retained by the register team, and an additional copy made for the participant’s GP.

The consent procedure was iteratively updated (following ethical approval) in the early optimisation stages of the register development. For example, the first version of the informed consent form included an option for the invitee to indicate that they did not wish to join the register. However, this option was removed following feedback from some invitees querying the apparent need to reply to the register team if they did not wish to join. The participant information sheet was also amended to highlight that no action would be required if an individual did not wish to join the register, and to reiterate that no information would be shared without consent. Ethics approval was also obtained to simplify the procedure for the participant by including a single consent form, a countersigned copy of which was returned to the participant.

Experience from the pilot phase of the register indicated that a number of individuals would seek to join the register after hearing about it via word-of-mouth. The protocol was therefore updated to allow “self-referrals” from individuals whose GP was not associated with the register. In this case, an invitation pack (on Imperial College London letter-headed paper) would be sent directly to the individual from the register team. This also included a data collection form to obtain the data which would otherwise be collected from the GP (see Data Collection section). Recruitment through this self-referral pathway relied on individuals self-reporting the absence of a dementia diagnosis.

Operational changes were also made to the register, including the inclusion of a barcode screening identifier to assist with the positive identification of individuals where the handwriting on the consent form was unclear. Manual printing and envelope stuffing was replaced by automatic production by a third party with NHS Information Governance accreditation (CFH Docmail, Radstock). This greatly increased the rate at which invitations could be printed and mailed.

During the pilot phase of the register development, practice administrative staff were asked to perform the search and prepare the invitation mail-out. However, as the register developed, the register team were able to provide step-by-step guidance with performing the searches and preparing the invitation mail-out through the third-party printing company. In all cases, practice clinical or administrative staff were able to review the list of potential invitees prior to the mail-out.

During the development of the register’s protocol, it was proposed that, approximately three weeks after the invitations were posted, a member of the register team would follow-up with a phone call to invitees who had not returned their consent forms. This would enable recipients’ questions to be answered and missing or lost consent forms to be re-sent. The ethics committee recommended that phone calls to individuals should be made by surgery staff, as sharing these details outside the clinical care team would compromise the individuals’ confidentiality. Therefore, all participating staff at the GP surgeries were trained in the aims and objectives of the register, were provided with a script approved by the ethics committee, and were prompted to questions that were likely to be asked by individuals. However, as it was not possible to monitor the calls, it was difficult to validate the quality and accuracy of the information provided and to ensure patients’ queries were answered satisfactorily. Feedback also suggested that availability of surgery staff, expected to carry out the task in addition to their usual workload, was variable. Furthermore, a review of the phone calls made to invitees at one surgery indicated that of 304 phone calls made by the surgery, 95 were answered and only six of the patients subsequently returned their consent form. Therefore, it became clear at an early stage of the register’s development that those individuals who had not responded in writing were unlikely to subsequently consent to join the register, and that the phone calls represented a poor use of resources. Therefore, follow-up phone calls were removed from the recruitment strategy.

### Data collection

Participants who agreed to join the CHARIOT register provided consent for their GP to share a minimum data set from their electronic health record with the register team. This included contact details (name, address, telephone number and email address), demographic details (date of birth, sex and ethnicity), along with the participant’s NHS number. No medical information was collected. Data were automatically extracted and encrypted at the practice by the register team, and transferred to a secure database at Imperial College London.

### Participant recruitment to research studies

The aim of the register is to aid recruitment to studies for the prevention of dementia and other late onset neurodegenerative conditions, and ethically approved studies are reviewed by a scientific review committee. Potential study participants can be identified based on age, gender and area of residence (based on distance to a study site). The CHARIOT register team contacts eligible individuals, inviting them to join the study–no data are shared with the other research teams, unless additional participant consent is obtained. The participant’s GP is not involved in this stage of recruiting into a follow-up research study, although individual study protocols may elect to inform the participant’s GP of their involvement.

### Data analysis

Descriptive data on the demographic profile of the register participants are reported. However, as data were only collected for consented individuals, it is not possible to directly compare the demographics of the CHARIOT cohort with the whole of the invited cohort, therefore these demographics are estimated from the National General Practice Profiles [27]. Age and gender profiles are compared against aggregate data from the practice profiles. As ethnicity data is only available from these profiles at a practice level, without additional detail on age and gender, we use a two-sample t-test to compare the practice-level ethnicity of the register participants with the profiles. For each practice we then calculate the normalised root mean square error of the difference between the actual and expected age/gender profile, and use Pearson’s correlation coefficient (r) to determine if the practices are equally representative, regardless of the ethnicity of its population.

Pearson’s correlation coefficient was used to examine the correlations between response rate and the demographic properties of the recruiting GP surgeries. We examined the correlations between response rate and: practice size (based on the list size); the age profile of the practice (based on the proportion of the list aged 65 years or older); the socioeconomic status of the practice population (measured by the Index of Multiple Deprivation–IMD); and the ethnicity of the practice population (indicated by the proportion of White patients registered at the practice).

## Results

The CHARIOT register has developed over a three year period, with three active recruitment periods between August–October 2011 (pilot phase), March–August 2012 (optimisation phase), and November 2012 –July 2014 (recruitment phase). During the initial pilot phase, three surgeries in North West London were approached to gauge the level of interest in the register, and a further ten surgeries participated in the optimisation phase. In the final recruitment phase, 110 surgeries from the wider west and central London geographic area joined the register.

From the 123 GP surgeries, 109,847 patients were invited to join the register. During the third recruitment period, a median of 4,690 individuals and a maximum of 7,482 individuals were invited each month. In total, 24,509 individuals consented to join the register, representing an overall response rate of 22.3%. Responses per surgery varied between 4.5% and 39.1% (median 20.2%). Of the individuals who joined the register, 53.3% were female, compared with an estimate of 52.8% from the National General Practice Profiles.

**Fig 1:**
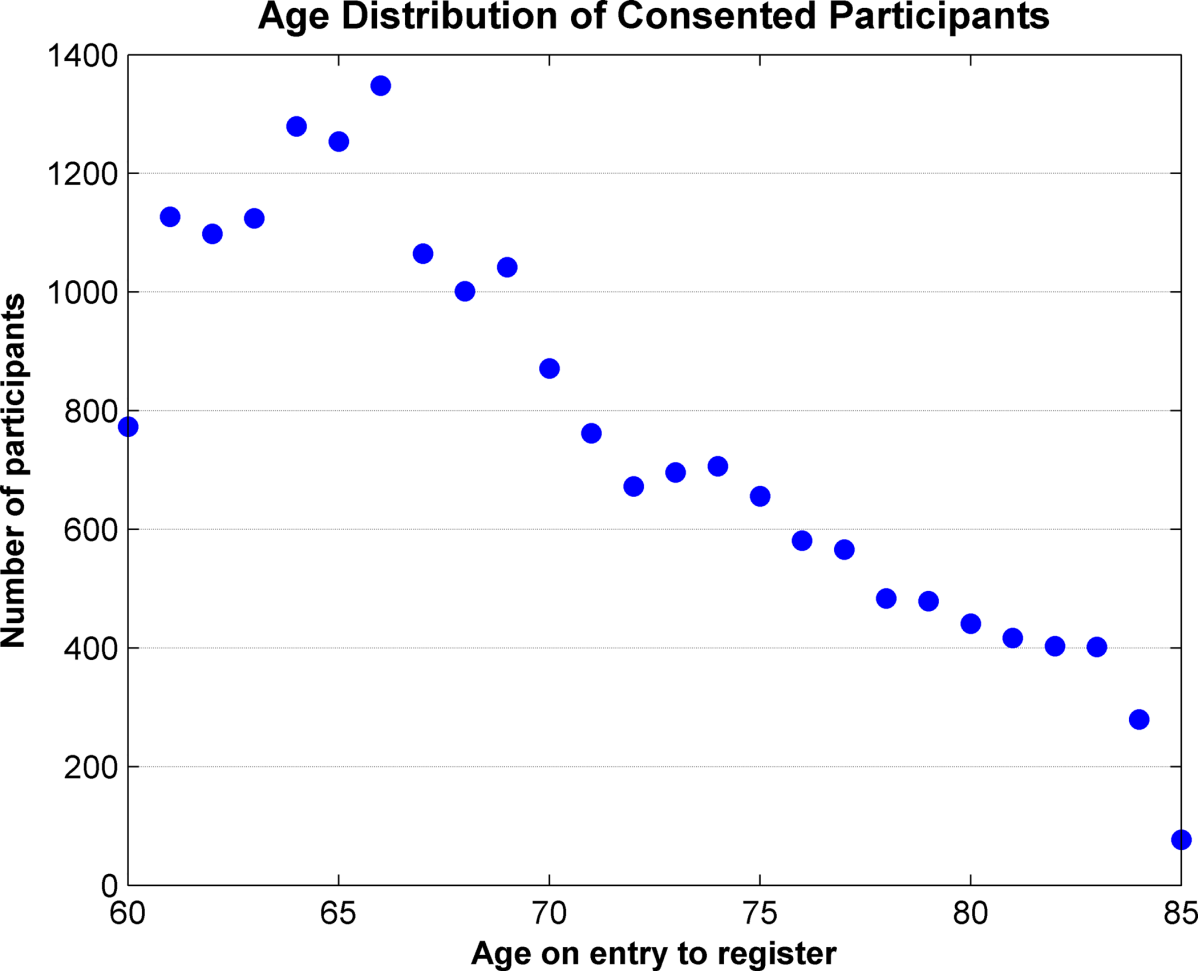
Age distribution of consented participants.

**Fig 2:**
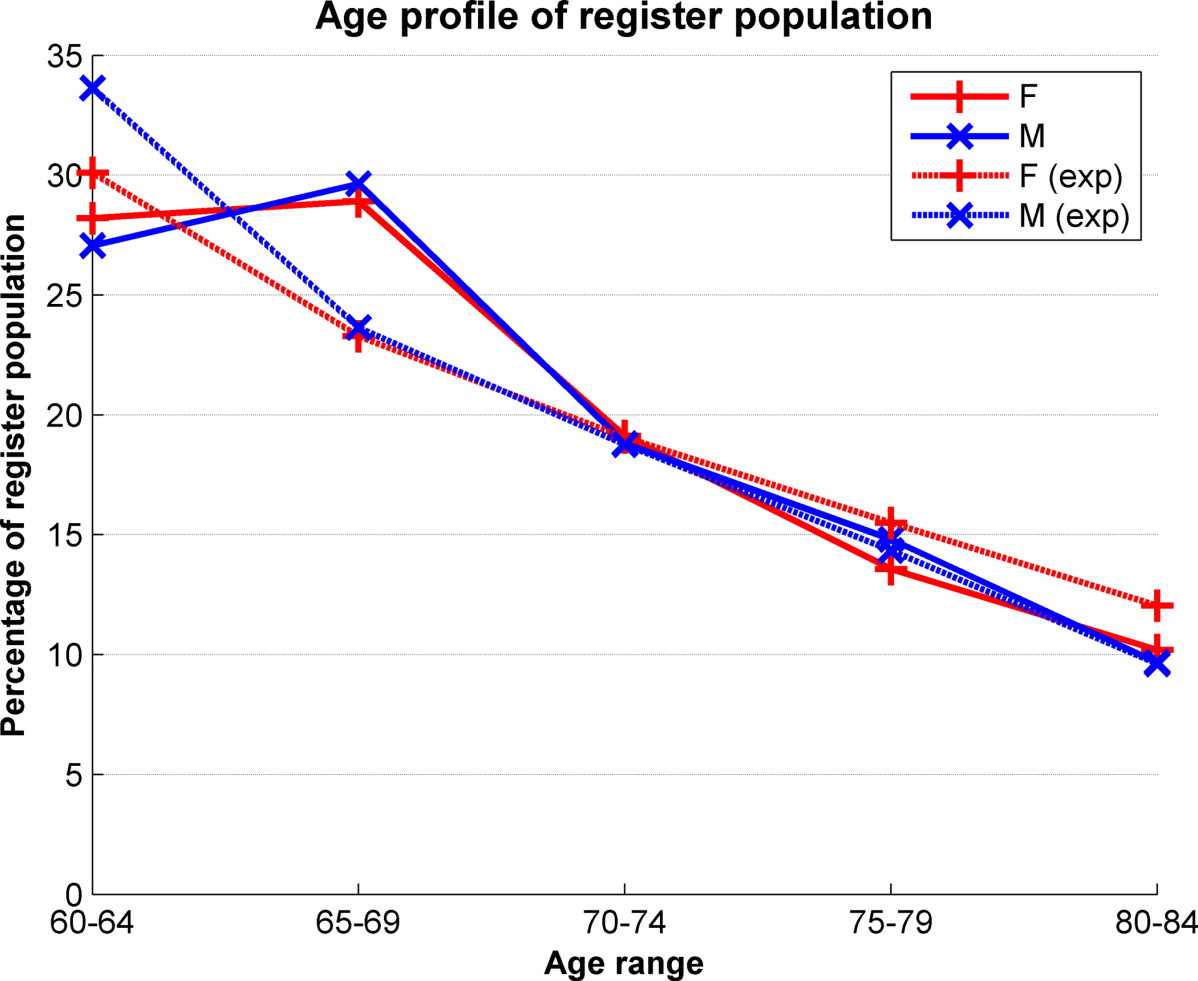
Response rate based on age and gender profiles. The solid lines represent the observed age distribution within the register population, and the dotted lines represent the expected profile based on the general practice profiles.

The median age of participants upon joining the register was 67 years, with a mode at 66 years as shown in Fig 1. Aggregating responses according to the age bands provided by the National General Practice Profiles allows an estimate of the response rate across the relevant age bands, as shown in Table 2. The response rate is also shown separately for female and male participants in Fig 2, and this shows the expected general decrease in the number of eligible participants as age increases. The response rates for both women and men appear to be slightly higher than expected in the 65–69 age group, otherwise the age profile of the register closely matches the invited cohort.

**Table 2:** Age profile of register compared to invited population.

Age Range	Estimated Proportion of Eligible Patients [27]	Proportion of Consented Participants
60–64	31.8%	27.5%
65–69	23.5%	29.1%
70–74	18.9%	18.9%
75–79	15.0%	14.1%
80–84	10.9%	9.9%

**Fig 3:**
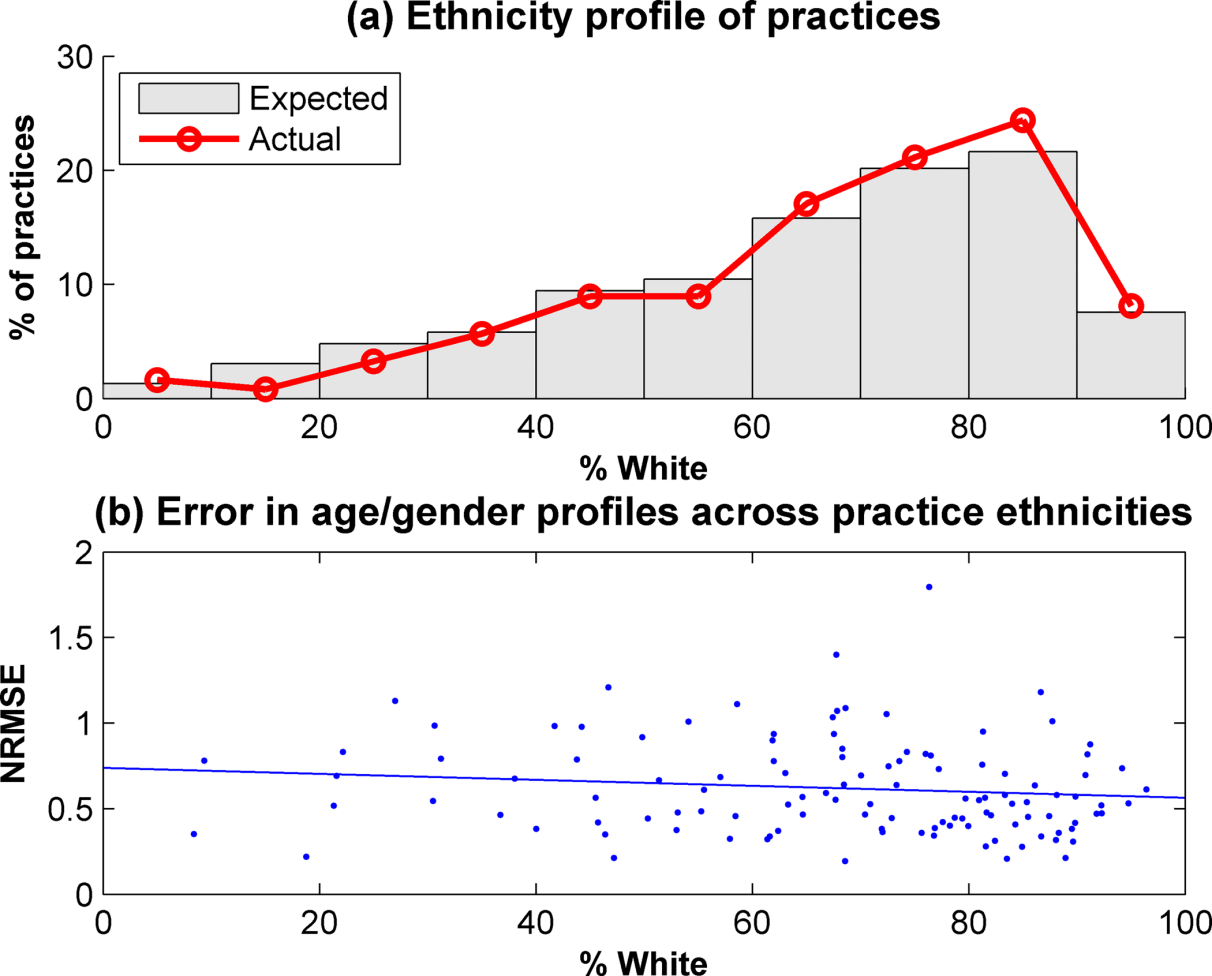
Ethnicity profiles of participating practices.

Ethnicity data were not available for 17.6% of the consented participants. There is also a lack of comprehensive ethnicity data available at a practice level, with only an approximation based on patient satisfaction surveys [27]. Therefore, it is difficult to assess how well the register population reflects the ethnic diversity of the eligible population. However, of the participants with ethnicity data recorded, 71.9% were White, compared to an estimate of 72.3% from the practice survey data. The ethnicities of the practices which recruited to the CHARIOT register were compared with the profiles of practices in the recruiting areas, as shown in Fig 3A. There was a slight over-representation of practices with larger White populations, although the difference between the CHARIOT practices and the overall group of practices was not significant (p = 0.220). The normalised root mean square error of the differences in expected and actual age/gender profiles within a practice are shown in Fig 3B. There is a slight trend towards practices with a larger White population having a smaller error metric, indicating a more representative age/gender profile, although this trend is not significant (p = 0.175).

**Fig 4:**
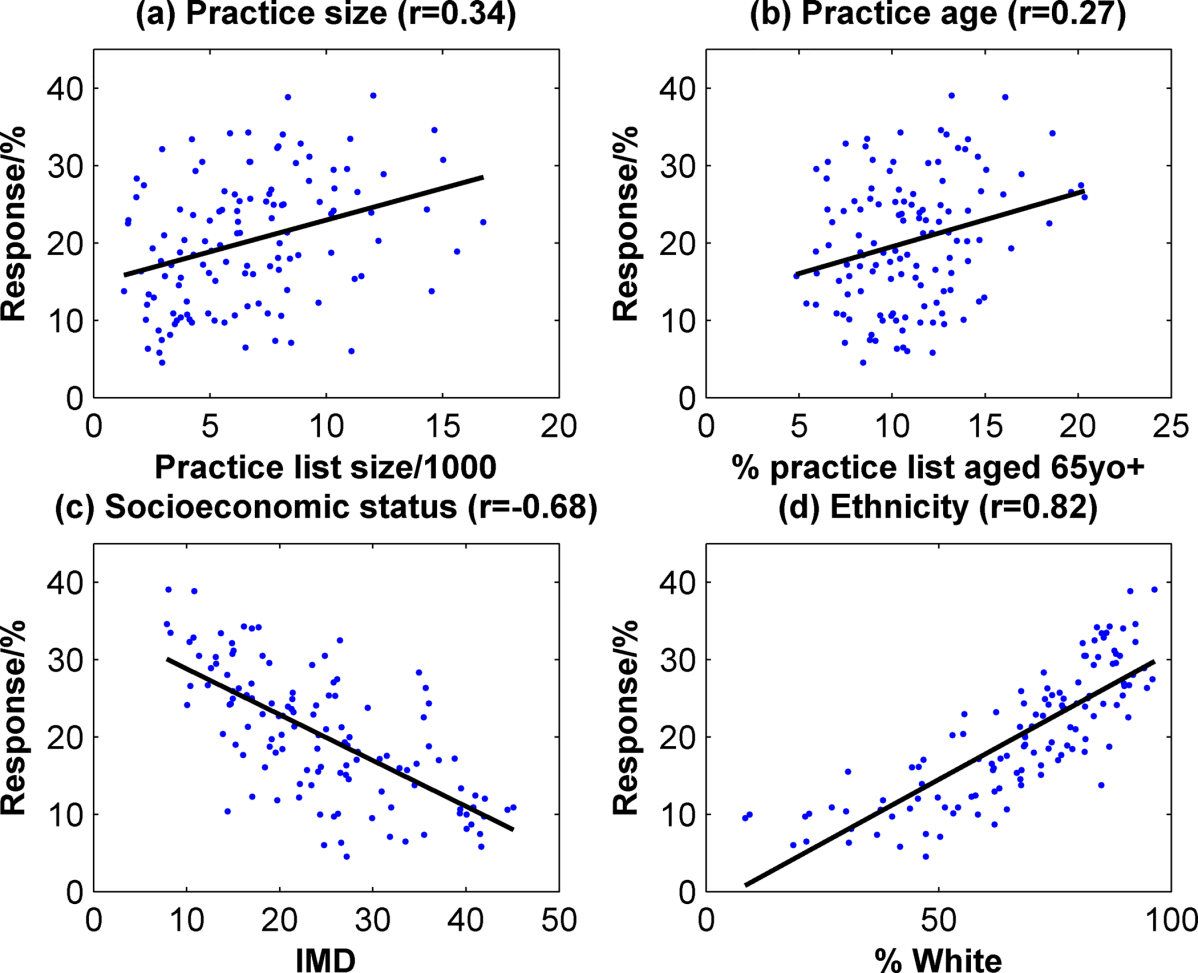
Correlations between response rate and practice metrics.

Based on these data, it appears that the register population provides a good representation of the wider population. However, as there was a large variation in the response rate (4.5–39.1%), we sought to understand the factors which may impact on response by examining the correlations between response rate and practice demographics–these data are shown in Fig 4. There was a slight trend towards larger practices having a higher response rate (r = 0.34, p<0.001), as well as those with an older population (r = 0.27, p = .0003). More significantly, practices with a higher IMD were correlated with a lower response (r = -0.68, p<0.001). Practices with a larger proportion of White patients also experienced a higher response rate (r = 0.82, p<0.001).

At the time of writing, three studies have been approved to recruit through the CHARIOT register, and these studies will be reported elsewhere as they follow independent clinical protocols. Preliminary data from the first study indicate a response rate of 40.9% of participants contacted about a telephone screening study elected to receive additional information, 44.4% of whom were subsequently eligible to join the study [28]. The main reason for exclusion was the requirement for individuals to self-report subjective complaints of memory problems, or other cognitive difficulties. Of those who enrolled in the study, 22.0% were identified as having abnormal cognition based on the AD8 Dementia Screening Interview [29] and the Rey Auditory Verbal Learning Test (RAVLT) [30].

## Discussion

The CHARIOT register is a novel primary care-based mechanism for recruitment of participants into research studies for the prevention of AD and other age-related neurodegenerative diseases. The register has brought together a multidisciplinary team to successfully enrol nearly 25,000 individuals in three years. A number of challenges in recruiting patients from primary care were identified, including ethical (for example, managing the process for individuals who do not wish to join the register) and operational challenges (for example, streamlining the process for mailing and addressing the challenges of follow-up phone calls).

A response rate of 22% indicates a good resonance with individuals wishing to join the register and is comparable to other established research registers. For example, the Scottish Health Research Register, a centralised register embedded within the health service, had a response rate of 13% from GP invitation letters [31], and the Hunter Medical Institute Research Register reported a response rate of 31% [32]. The results from the first study to recruit from the register show a strong response rate (41%). This provides a compound response rate, accounting for recruitment into the register and then into study screening, of 9.1%. Although this figure appears low, it compares favourably with other studies recruiting directly through postal invitations [33]. The high response rate from the register into a study indicates the power of such an established register for other researchers, being able to rapidly recruit large numbers of participants into studies. Importantly such an approach does not burden participants’ GPs with recruitment into studies. Participants who are on the register and may not fulfil inclusion criteria for one study may however be eligible for another–therefore we expect that the total recruitment yield of the register to be ultimately much higher than 9%. Of those individuals who were screened to join the study, 44% were eligible based on a self-report of early stage symptoms, such as memory or other cognitive problems. Future studies aimed at a true pre-symptomatic population are likely to see higher proportions of eligible individuals.

The basic inclusion and exclusion criteria allow us to recruit a representative sample of the population aged 60–85, excluding only those with an existing diagnosis of dementia. Using this model there is scope to set up this or similar registers throughout the UK and internationally. This would allow greater access to trials and greater opportunity for researchers to recruit a highly motivated and willing population. Such expansion could be supported by the development of a website or mobile/tablet app to improve the ability to communicate with potential and current participants.

We recognise that this approach has limitations and possible deficiencies. We have recruited a large number of individuals; however, it is not known how many of the participants may already have undiagnosed problems with cognition. Due to the age range of participants invited to join the register, we would expect some participants to demonstrate early signs of the onset of dementia, and anecdotal evidence from telephone calls received by the register office suggests that a large number of individuals are worried about their memory. However it is not clear to what extent this is related to the increasing awareness of the high prevalence of dementia in the older population or to normal age-related memory loss. The mixture of participants at different stages of disease progression, or none, will allow different prevention studies to target different disease stages, with follow-on studies screening for specific eligibility criteria. The first study to recruit from the register indicated 22% of the screened participants with subjective complaints may have abnormal cognition, therefore enriching the register with other clinical data, including biomarkers, may be a useful strategy [5]. The collection of additional data may allow improved characterisation of the register population, allowing for stratification based on dementia risk as a pre-screening tool to select appropriate candidates for specific inclusion and exclusion criteria of future studies.

To improve the quality of data available, consent may be gathered during recruitment into the register, allowing collection of additional medical or demographic data from the GP record. However, this is likely to affect the recruitment rate as individuals may be happy for collection of the minimal contact and demographic details, but not of their medical data. Extraction of additional demographic or socioeconomic data from the medical record is also likely to be hampered by an underlying lack of available data. Our analysis found 17.6% of individuals joining the register did not have their ethnicity recorded in the medical record, although this compares favourably with a national review of anonymised centrally-held records which found this information was not available for 53.8% of patients nationwide [34]. 86% of patients in England have a smoking status recorded in the health record [26], however other lifestyle data are not routinely collected in general practice in the UK. For example, a review of the collection of data on alcohol consumption found only 9% of newly registered patients were recorded as completing a validated screening test [35]. Socioeconomic data at an individual level is unlikely to be collected, and other studies have also used area-level aggregate data from other sources [36]. Additional consent may be sought for collection of these additional data directly from register participants, or for phenotypic characterisation of a sample of the register population by conducting a battery of cognitive and other tests (for example, neuroimaging), or collection of biological samples.

In order to address some of the missing data, we have implemented a process for opportunistically collecting missing ethnicity information from participants who have direct contact with a register team member, principally by telephone. We also have a framework in place for secondary studies which collect ethnicity data, with appropriate participant consent, to share these data back to the register. Using these additional ethically approved approaches, we are progressively building a more complete dataset.

Complete demographic data are not available to provide a comprehensive analysis of responders compared to non-responders, however, based on practice-level data the register appears to match the expected age, gender and ethnicity profiles of the wider population. There was a slight trend towards larger practices having higher response rates, possibly due to the presence of additional resources available to indirectly support recruitment. Larger practices are likely to have additional administrative staff who can support the direct recruitment tasks, rather than relying on the involvement of practice managers or general practitioners themselves, as well as having more developed organisational procedures in place [37]. Lower response rates were associated with practices with higher IMDs, and larger Black and Minority Ethnic populations. Although a representative sample was obtained overall, this highlights the challenges of recruiting participants from these groups. Due to the cultural and linguistic diversity in the recruitment region, further recruitment strategies may be augmented by including recruitment material in alternative languages.

The CHARIOT register has been successfully established, consisting of a large cohort of individuals interested in participating in dementia prevention research. Response rates of individuals consenting to join the register indicate interest and support for a research register and for participation in research for the prevention of age-related neurodegenerative diseases and dementia. The simplicity of this approach means that this system is easily scalable, increasing the availability of participants and the ease of recruiting participants for research studies into the prevention of dementia.

## Supporting Information

S1 File Exclusion Criteria. (DOCX)
