# Supplementary material for: Development of the CHARIOT Research Register for the Prevention of Alzheimer’s Dementia and Other Late Onset Neurodegenerative Diseases
Source: PLoS One. 2015 Nov 23;10(11):e0141806. doi: 10.1371/journal.pone.0141806 (PMC4657961; doi:10.1371/journal.pone.0141806)
Supplement: S1 File — (DOCX) [file pone.0141806.s001.docx]

**S1 Exclusion Criteria.** Standard read code terms used to denote a diagnosis of dementia. Terms shown in bold are “parent” terms, and those in italics are implicit terms derived from these parent terms.

NOS = not otherwise specified

| **Term** |
| --- |
| **Senile and presenile organic psychotic conditions** |
| *Uncomplicated senile dementia* |
| *Presenile dementia* |
| *Uncomplicated presenile dementia* |
| *Presenile dementia with delirium* |
| *Presenile dementia with paranoia* |
| *Presenile dementia with depression* |
| *Presenile dementia NOS* |
| *Senile dementia with depressive or paranoid features* |
| *Senile dementia with paranoia* |
| *Senile dementia with depression* |
| *Senile dementia with depressive or paranoid features NOS* |
| *Senile dementia with delirium* |
| *Arteriosclerotic dementia* |
| *Uncomplicated arteriosclerotic dementia* |
| *Arteriosclerotic dementia with delirium* |
| *Arteriosclerotic dementia with paranoia* |
| *Arteriosclerotic dementia with depression* |
| *Arteriosclerotic dementia NOS* |
| *Other senile and presenile organic psychoses* |
| *Senile or presenile psychoses NOS* |
| **Other alcoholic dementia** |
| *Chronic alcoholic brain syndrome* |
| **Drug-induced dementia** |
| **Dementia in conditions classified elsewhere** |
| **Dementia in Alzheimer's disease** |
| *Dementia in Alzheimer's disease with early onset* |
| *Dementia in Alzheimer's disease with late onset* |
| *Dementia in Alzheimer's dis, atypical or mixed type* |
| *Dementia in Alzheimer's disease, unspecified* |
| **Vascular dementia** |
| *Vascular dementia of acute onset* |
| *Multi-infarct dementia* |
| *Subcortical vascular dementia* |
| *Mixed cortical and subcortical vascular dementia* |
| *Other vascular dementia* |
| *Vascular dementia, unspecified* |
| **Dementia in other diseases classified elsewhere** |
| *Dementia in Pick's disease* |
| *Dementia in Creutzfeldt-Jakob disease* |
| *Dementia in Huntington's disease* |
| *Dementia in Parkinson's disease* |
| *Dementia in human immunodeficiency virus [HIV] disease* |
| *Lewy body dementia* |
| *Dementia in other specified diseases classified elsewhere* |
| *Unspecified dementia* |
| **Delirium superimposed on dementia** |
| **Alzheimer's disease** |
| *Alzheimer's disease with early onset* |
| *Alzheimer's disease with late onset* |
| **Pick's disease** |
| **Senile degeneration of brain** |
| **Lewy body disease** |
